# Prevalence, distribution, and associated factors of suicide attempts in young adolescents: School-based data from 40 low-income and middle-income countries

**DOI:** 10.1371/journal.pone.0207823

**Published:** 2018-12-19

**Authors:** Xiang Liu, Yi Huang, Yuanyuan Liu

**Affiliations:** 1 Department of Health and Social Behavior, School of Public Health, Sichuan University, Chengdu, Sichuan Province, China; 2 Mental Health Center, West China Hospital, Sichuan University, Chengdu, Sichuan Province, China; 3 Department of Epidemiology and Health Statistics, School of Public Health, Sichuan University, Chengdu, Sichuan Province, China; Chiba Daigaku, JAPAN

## Abstract

Suicide attempts are the most important known predictor of death by suicide. The aim of this study is to examine the prevalence, distribution, and associated factors of suicide attempts among young adolescents in 40 low-income and middle-income countries. We used data from the Global School-Based Student Health Survey (2009–2013) and a nationally representative study in China (2010), which are school-based surveys of students primarily aged 12–18 years that assess health behaviors using an anonymous, standardized, self-reported questionnaire. We calculated the prevalence of suicide attempts in young adolescents from 40 low-income and middle-income countries using the surveys. Multilevel logistic models were used to estimate the associations between suicide attempts and potential risk factors, adjusting for gender, age, school and survey year. Results show that the mean 12-month prevalence of suicide attempts was 17.2%, ranging from 6.7% in Malaysia to 61.2% in Samoa. The overall prevalence of suicide attempts was higher for girls than for boys (18.2% vs 16.2%, *P*<0.05). Among the suicide attempts, the proportion of suicide attempts with a plan was higher for girls than for boys (62.7% vs 53.2%, *P*<0.05). Both the prevalence of suicide attempts and the proportion of suicide attempts with a plan increased with age. Factors associated with suicide attempts included poor socioeconomic status, history of bullying, loneliness and anxiety, tobacco and alcohol use, and weak family and social relationships. In conclusion, suicide attempts are frequent among young adolescents in low-income and middle-income countries. Girls and older adolescents tend to make suicide attempts with a plan. The data demonstrate the need to strengthen suicide intervention and prevention programs for young adolescents in low-income and middle-income countries.

## Introduction

Suicide is a serious public health problem that affects children and adolescents[1]. The World Health Organization (WHO) indicated that suicide is the second leading cause of death for youths between the ages of 15 and 29[2], particularly girls 15–19 years old, for whom suicide is the leading cause[3]. For each completed suicide, it is estimated that 100–200 adolescents make a suicide attempt[4]. More importantly, a prior suicide attempt is the most important known risk factor for death by suicide[5,6]. Individuals who have previously attempted suicide are at a much higher risk of dying by suicide compared with those who have never attempted suicide[7,8]. Additionally, some studies have suggested that suicide attempts predict various problems, including physical health problems, mental disorders, interpersonal difficulties, harming others and greater treatment service utilization[9–11].

Suicide attempts often emerge in adolescents and are prevalent among this age group. The prevalence of suicide attempts in adolescent varies widely by region, age, gender, ethnicity and other factors. Across 17 European countries, the lifetime prevalence of suicide attempts among students aged 15–16 years varied from 4.1% to 23.5%[12]. The 2013 Youth Risk Behavior Survey of students in the United States demonstrated that 5.4% of boys and 10.6% of girls had attempted suicide[13]. While most of the data come from Europe and North America, these developed countries generally have sophisticated youth risk behavior surveillance systems that can monitor youth risk behaviors, including suicide attempts. Compared with developed countries, studies on the prevalence and determinants of adolescent suicide attempts in low- and middle-income countries (LMICs) are limited, as available data on suicidal behaviors are often lacking in LMICs. In reality, LMICs bear most of the global suicide burden, with an estimated 75% of all suicides occurring in these countries[2], as the majority of the world’s population live in LMICs. A growing number of studies assessing adolescent suicide attempts have been conducted in several LMICs, including China, Mexico, and Ghana[14–16]. Although studies from individual countries have expanded knowledge in regard to adolescent suicide attempts in LMICs, inconsistencies in the definition of a suicide attempt, data collection methods and measured recall time-points make it difficult to compare the prevalence and risk factors of adolescent suicide attempts across different countries.

The Global School-Based Student Health Survey (GSHS) is a collaborative surveillance project designed to assess adolescent health risk behaviors and exposure, supported by the WHO[17]. GSHS data have been applied to study the prevalence of adolescent suicide attempts and related risk factors in individual LMICs and Western Pacific Island countries[16,18–20]. Notably, two cross-national studies explored adolescent suicidal behaviors among all the low- and middle-income countries using the GSHS completed between 2003 and 2012[21,22]. However, both studies compiled only the prevalence of suicidal ideation without suicide attempts among adolescents. In 2009, a question about suicide attempts was added to the GSHS for most countries. It is essential to monitor the distribution of suicide attempts in young adolescents to provide important information for the development of suicide prevention strategies. In this study, we estimate the prevalence of suicide attempts among young adolescents and examine the factors associated with suicide attempts in 40 LMICs.

## Methods

### Data sources

We used data on suicide attempts from the GSHS completed between 2009 and 2013 for all countries. This survey was developed by the WHO in collaboration with UNICEF, UNESCO, and UNAIDS, with technical assistance from US Centers for Disease Control and Prevention (CDC). The GSHS used a standardized sample selection process, a two-stage random cluster sampling of schools and classes to select eligible participants mainly aged 12–18 years old from each country[23]. All GSHS versions were approved by institutional review boards or ethics committees and the Ministry of Health or Education of each country. Written or verbal consent was also obtained from the students and their parents.

We included countries for which survey data on suicide attempts and potential risk factors were available. Countries with missing reports of these items were not selected. Finally, we included 39 countries from five WHO regions (Africa, the Americas, Eastern Mediterranean, Asia and Western Pacific). The survey dates ranged from 2009, the first year that the suicide attempt query was added to the questionnaire, to 2013, the latest year with publicly available data at the time of this study. Table 1 shows the characteristics of the included surveys from the GSHS.

**Table 1:** Survey characteristics of the GSHS according to country, 2009–2013.

Country	Survey year	Sample size	Response rate (%)	Boys (%)
**Africa (6 countries)**				
Benin	2009	2649	99.7	65.0
Ghana	2012	3543	99.0	53.8
Malawi	2009	2212	99.1	46.9
Mauritania	2010	1976	98.0	47.1
Namibia	2013	4410	98.5	47.2
Swaziland	2013	3612	97.4	47.5
**Americas (17 countries)**				
Antigua and Barbuda	2009	1216	98.8	46.6
Argentina	2012	27778	98.4	47.7
Bahamas	2013	1340	97.9	46.4
Belize	2011	1968	98.7	47.5
Bolivia	2012	3438	98.5	50.6
British Virgin Islands	2009	1584	99.1	44.9
Costa Rica	2009	2653	99.5	48.3
Dominica	2009	1599	99.1	43.7
Guatemala	2009	5419	99.2	45.5
Honduras	2012	1715	99.5	48.2
Jamaica	2010	1570	99.2	48.9
Peru	2010	2842	99.0	48.8
Saint Kitts and Nevis	2011	1714	98.8	44.0
Salvador	2013	1858	99.5	53.8
Suriname	2009	1673	98.9	51.1
Trinidad and Tobago	2011	2621	98.9	54.5
Uruguay	2012	3455	99.4	46.4
**Eastern Mediterranean (6 countries)**				
Iraq	2012	1980	98.9	56.4
Kuwait	2011	2650	98.9	50.3
Lebanon	2011	2248	99.6	46.7
Morocco	2010	2830	97.7	52.5
Palestine	2010	4441	97.3	47.3
United Arab Emirates	2010	2551	99.1	42.0
**Asia (5 countries)**				
Cambodia	2013	3783	99.6	47.2
Malaysia	2011	25421	99.9	50.0
Mongolia	2013	5301	99.3	46.8
Philippines	2011	5212	99.8	43.2
China*	2010	8820	-	50.5
**Western Pacific (6 countries)**				
Kiribati	2011	1555	98.6	43.5
Niue	2010	134	100.0	59.7
Samoa	2011	2303	93.7	41.2
Solomon Islands	2011	1293	97.1	52.0
Tuvalu	2013	899	98.3	48.5
Vanuatu	2011	1014	100.0	44.2

* China is one of the important countries in Asia, and it has large number of young adolescents, so we included China in our study. Because GSHS data in China were collected in 2003, we used data from a large sample size study performed in 2010 in China[24]. This research is part of a program for youth health risk behavior assessments in China, which was supported by China's Ministry of Science and Technology. The research was a school-based survey of suicidal behaviors among students mainly aged 12–18 years. The selection of eligible students and the questions used to assess suicidal behaviors were similar between this study and the GSHS.

### Measures

The GSHS consists of several questionnaire modules that address the leading causes of morbidity and mortality among children worldwide, including tobacco use, alcohol and drug use, violence and unintentional injuries, mental health and sexual behaviors[23]. There are no published studies demonstrating the validity or reliability of the GSHS. However, many items included on the GSHS adopted from other youth risk behavior surveillance systems such as the YRBS, the GYTS and the Health Behavior in School-aged Children (HBSC) survey. Further, there is evidence that reporting health risk behaviors in students show good reliability[25].

In the GSHS, suicide attempts were assessed with the question: “During the past 12 months, how many times did you actually attempt suicide?”. Suicide attempts were defined as at least 1 attempt in the past 12 months. To quantify the type of suicide attempt (suicide attempt with a plan and without a plan), the suicidal plan was assessed with the question “During the past 12 months, did you make a plan for how you would attempt suicide?”.

We selected the potential risk factors for suicide attempts on the basis of previous research. The determinants included substance use (cigarette smoking and alcohol use), bullying victimization, physical activity, mental condition (anxiety and loneliness) and social support (close friends and parental support). Given that the socioeconomic status of the young adolescents was not included in the GSHS, we assessed a proxy variable based on the frequency of going hungry. The relevant questions for the variables and coding are indicated in Table 2.

**Table 2:** GSHS questions used in the analysis of adolescent suicide attempts.

Variable	Question	Values
Suicide attempt	During the past 12 months, how many times did you actually attempt suicide?	0 = No 1 = Yes
Suicide planning	During the past 12 months, did you make a plan for how you would attempt suicide?	0 = No 1 = Yes
Lack of food	During the past 30 days, how often did you go hungry because there was not enough food in your home?	1 = Never/Rarely/Sometimes 2 = Most of the time/Always
Bullying	During the past 30 days, on how many days were you bullied?	1 = No (0 days) 2 = Yes (1 or more days)
Loneliness	During the past 12 months, how often have you felt lonely?	1 = Never/Rarely/Sometimes 2 = Most of the time/Always
Anxiety	During the past 12 months, how often have you been so worried about something that you could not sleep at night?	1 = Never/Rarely/Sometimes 2 = Most of the time/Always
Parental support	During the past 30 days, how often did your parents or guardians understand your problems and worries?	1 = Never/Rarely/Sometimes 2 = Most of the time/Always
Close friends	How many close friends do you have?	1 = No (none) 2 = Yes (1 or more)
Alcohol use	During the past 30 days, on how many days did you have at least one drink containing alcohol?	1 = No (0 days) 2 = Yes (1 or more days)
Cigarette smoking	During the past 30 days, on how many days did you smoke cigarettes?	1 = No (0 days) 2 = Yes (1 or more days)

We used the GDP per capita for each country corresponding to the survey year as reported by the World Bank for most countries and reported by the Index Mundi for the few countries for which GDP per capita was not listed in the World Bank list.

### Statistical analysis

All data were weighted based on a random cluster sampling design to provide nationally representative estimates for each country. We used the Complex Samples module in SPSS version 21.0 to estimate the weighted prevalence (with corresponding 95% CIs) by country and gender. As significant heterogeneity was noted between countries (I^2^>95%), a random effects meta-analysis model was applied for the following analyses: (1) to estimate the regional and total prevalence of suicide attempts; (2) to test the differences in the prevalence of suicide attempts between genders and age groups; and (3) to test the differences in the proportion of suicide attempt type between genders and age groups. According to the random cluster sampling, multilevel logistic regression models based on three levels (country, school and individual level) were employed to estimate the associations between the determinants and suicide attempts. We initially fit an empty model with no explanatory variables (model 1) that was used to estimate the intra-class correlation coefficient (ICC) [26]. The ICC is interpreted as the proportion of the total variance that can be attributed to the higher level in the analysis. Therefore, the ICC indicated the degree to which suicide attempts clustered within countries and schools. Subsequently, we introduced all potential explanatory variables in the model after adjusting for age, gender, school, country and survey year (model 2). A two-sided *P* value of less than 0.05 was considered statistically significant.

## Results

We included the data from 146460 young adolescents (48.4% boys) aged 12–18 years from the GSHS and 8820 Chinese young adolescents (50.5% boys) aged mainly 12–18 years from a nationally representative study. The median sample size per country (for the GSHS data) was 2303.

Table 3 shows the 12-month prevalence of suicide attempts among young adolescents by WHO region and gender. In total, 17.2% of the assessed young adolescents reported having made a suicide attempt in the past year. The prevalence of suicide attempts was highest in the Western Pacific region (28.6%) and lowest in the Asian region (8.9%). The overall prevalence of suicide attempts was higher for girls than for boys (18.2% vs 16.2%, *P*<0.05). However, the gender difference was not consistent across different regions. The prevalence of suicide attempts was higher in girls in the Americas and Asian regions, while it was higher in boys in the Western Pacific region (*P*<0.05, Table 3).

**Table 3:** Prevalence of suicide attempts in young adolescents by region and gender.

Region	Boys	Girls	Total
Africa	20.6% (15.3–25.9)	20.7% (15.7–25.7)	20.6% (15.5–25.7)
Americas	11.7% (10.2–13.2)	16.8% (14.8–18.7) *	14.3% (12.7–15.9)
Eastern Mediterranean	15.4% (12.2–18.5)	16.7% (14.1–19.3)	16.0% (13.2–18.7)
Asia	7.8% (5.6–10.0)	9.8% (6.2–13.4)*	8.9% (5.9–11.9)
Western Pacific	31.2% (12.6–49.9)	26.8% (4.2–49.4)*	28.6% (10.0–47.3)
Total	16.2% (13.7–18.7)	18.2% (15.5–20.9) *	17.2% (14.7–19.7)

*P<0.05 for the difference between genders.

The prevalence of suicide attempts ranged from 6.7% in Malaysia to 61.2% in Samoa. Of the 40 countries, 33 (82.5%) countries exceeded a 10% suicide attempt prevalence rate (S1 Table).

Table 4 shows the prevalence of suicide attempts and the proportion of suicide attempt type by gender and age. Among the suicide attempts, girls were more likely to report suicide attempts with a plan than were boys (62.7% vs 53.2%, *P*<0.05). Across age groups, adolescents aged 16 years or older tended to make suicide attempts more than adolescents aged 12–13 years in most countries (*P*<0.05, S2 Table). The overall prevalence of suicide attempts was highest for those aged 16 years or older and lowest for those aged 12–13 years (20.1% vs 15.2%, *P*<0.05). Among the suicide attempts, adolescents aged 16 years or older were more likely to make suicide attempts with a plan than were adolescents aged 12–13 years (61.2% vs 54.1%, *P*<0.05).

**Table 4:** Prevalence of suicide attempts and proportion of suicide attempt type by gender and age.

Characteristics	Suicide attempt	Type of suicide attempt	
		Suicide attempt with a plan	Suicide attempt without a plan
Gender			
Boys	16.2% (13.7–18.7)	53.2% (49.9–56.5)	46.8% (43.5–50.1)
Girls	18.2% (15.5–20.9) *	62.7% (60.1–65.2)*	37.3% (34.8–39.9) *
Age			
12–13 years	15.2% (13.0–17.4)	54.1%(50.7–57.5)	45.9%(42.5–49.3)
14–15 years	17.5% (14.9–20.1) ^▲^	59.7%(57.5–61.9)^▲^	40.3%(38.1–42.5) ^▲^
16 years or older	20.1% (17.1–23.1) ^▲^	61.2%(58.3–64.1)^▲^	38.8%(35.9–41.7) ^▲^

* *P*<0.05 for the difference between genders.

^▲^*P*<0.05 for the difference between adolescents aged 12–13 years and the other age group.

Table 5 shows the results of multilevel logistic regression analyses of suicide attempts. In the empty model (model 1), the ICC was estimated to be 0.090 at the country level and 0.048 at the school level, indicating that 9.0% and 4.8% of the variance in suicide attempts could be attributed to country and school level, respectively. Model 2 indicated that lack of food (*OR* = 1.33, 95%*CI*: 1.23–1.44), being bullied (*OR* = 2.17, 95%*CI*: 2.07–2.27), loneliness (*OR* = 2.09, 95%*CI*: 1.97–2.21), anxiety (*OR* = 2.10, 95%*CI*: 1.97–2.23), cigarette smoking (*OR* = 2.13, 95%*CI*: 2.02–2.26) and alcohol use (*OR* = 1.66, 95%*CI*: 1.57–1.75) remained risk factors for suicide attempts after adjusting for other factors. Having close friends (*OR* = 0.61, 95%*CI*: 0.56–0.65) and parental support (*OR* = 0.72, 95%*CI*: 0.69–0.76) were significant protective factors for suicide attempts. There was no association between the prevalence of suicide attempts and the country’s per capita GDP (*P*>0.05).

**Table 5:** Multilevel logistic regression analyses of the factors associated with suicide attempts.

	Model 1		Model 2*		
Variables	Coefficient (SE)	*P*	Coefficient (SE)	*OR* (95% *CI*)	*P*
Fixed effect					
GDP per capita			-0.019(0.0015)	0.98(0.95–1.01)	0.209
Lack of food (Most of the time/Always)			0.285(0.0401)	1.33(1.23–1.44)	<0.001
Being bullied (Yes)			0.774(0.023)	2.17(2.07–2.27)	<0.001
Loneliness (Most of the time/Always)			0.736(0.0292)	2.09(1.97–2.21)	<0.001
Anxiety (Most of the time/Always)			0.740(0.0313)	2.10(1.97–2.23)	<0.001
Parental support (Most of the time/Always)			-0.326(0.0237)	0.72(0.69–0.76)	<0.001
Close friends (Yes)			-0.501(0.0394)	0.61(0.56–0.65)	<0.001
Cigarette smoking (Yes)			0.758(0.0288)	2.13(2.02–2.26)	<0.001
Alcohol use (Yes)			0.506(0.0266)	1.66(1.57–1.75)	<0.001
Random effect					
Level 3: Country	0.343(0.082)	<0.001	0.333(0.122)		0.006
Level 2: School	0.185(0.013)	<0.001	0.148(0.016)		<0.001

*Model 2 was adjusted for age, gender, school, country and survey year

## Discussion

Although the prevalence of suicide attempts varied between countries, suicide attempts are a common problem in LMICs among adolescents aged 12–18 years. In the present study, the 12-month prevalence of suicide attempts was 17.4% among adolescents, which is higher than the prevalence reported in most studies of developed countries, including a study across 17 European countries (10.5%)[12], the 2013 Youth Risk Behavior Survey of students in the United States (8.0%)[13] and a review of 128 studies from mostly developed countries (9.7%)[27]. Compared with the prevalence of suicide attempts among adults in developing countries based on the WHO World Mental Health Surveys[28], the prevalence of suicide attempts among adolescents in our study was much higher (0.3% vs 17.4%, respectively). These data indicate that suicide attempts among adolescents is an issue of major concern in LMICs.

The Western Pacific region exhibited the highest overall prevalence of suicide attempts. The three countries with the highest prevalence rates were Western Pacific countries, i.e., Samoa (61.2%), Solomon (33.6%) and Kiribati (31.5%). Several studies have demonstrated a significant increase in suicidal behavior in Pacific Island countries, especially among youths[29,30]. The reasons for this high prevalence may be explained by societal transitions from traditional to modern resulting in intergenerational conflict, the rise of the nuclear family, direct and indirect suicide contagion, and the co-occurrence of mental disorders[31,32]. The prevalence of suicide attempts in the African region was also considerable (20.6%), particularly for Benin (28.2%), Ghana (26.4%), and Namibia (25.6%), which followed the highest three Western Pacific countries. This may be partly attributed to food insecurities, political instability and the high prevalence of AIDS[33,34]. The high levels of suicide attempts in these regions and countries point to the need for increased attention in both research and policy.

Gender differences in suicide attempts were not consistent across different regions, especially in the Western Pacific region, where boys showed a higher prevalence of suicide attempts than did girls, contrary to the conclusions of most studies in developed countries. The prevalence of suicide attempts was higher for girls than boys in the Americas and Asia; however the prevalence of the girl-to-boy ratio in most of the countries was lower than that of high-income countries, which is approximately 2:1 or 3:1[12,13,35,36]. These findings implied that gender contributes less to suicide attempts in LMICs than in developed countries. When comparing the type of suicide attempt between genders, we found that the proportion of suicide attempts with a plan was greater for girls than for boys. This might be partly explained by higher levels of depression and lower levels of assertiveness in girls, which are associated with planned suicide attempts[37,38]. It is noteworthy that more than half of the suicide attempts were a planned suicide attempt. Several studies have documented that people who plan suicide attempts tend to choose more lethal suicide methods and, thus, have a higher risk of more severe medical consequences than those who implement impulsive suicide attempts without a plan[39,40]. Given that a suicide plan is developed prior to the actual suicidal behavior, this suggests the importance of identifying a suicide plan early.

In our study, older adolescents were more likely to report suicide attempts than were younger adolescents. This was consistent with the findings of Cui et al[41], yet inconsistent with several studies that reported that the likelihood of suicide attempts decreased in an inverse proportion to age among adolescents[15,42]. The differences in suicide attempts across age groups may be the result of interactions between sociocultural, psychological, developmental and family environmental factors[43]. The present study found that the proportion of suicide attempts without a plan was higher for younger adolescents than for older adolescents, which might indicate that impulsive suicide attempts were more prevalent in younger adolescents. Reasons for this finding may be that younger adolescents who engage in suicide attempts are more ambivalent about willing to die at the time of the act and that some suicidal behaviors are impulsive responses to acute psychosocial stressors[2]. Restricting access to the means of suicide is crucial for suicide prevention, particularly in the cases of impulsive suicide attempts, because this restriction provides an opportunity for these individuals to think clearly about what they are about to do, and this may help them give up suicidal behavior[2]. However, means-restriction policies require an understanding of the method preferences of different groups. Unfortunately, data on the methods used in suicide attempts among adolescents are quite limited. Hence, further studies on suicide attempts addressing suicide methods should be conducted.

The results of the multilevel logistic regression models indicated that a significant amount of the variation in suicide attempts can be attributed to differences between countries and schools. Schools represent an important social context for adolescents. It has been suggested that suicidal behaviors may cluster within schools as a result of interpersonal interactions with peers who are suicidal[44]. The contextual effect of a country’s aggregate level on suicide attempts is notable, although we found no association between the prevalence of suicide attempts and the country’s per capita GDP. This indirectly suggests that the prevalence of suicide attempts among adolescents depends on multiple non-economic factors, such as ethical values, religious beliefs, media reports and mental health education. The concept of influence of these factors needs to be taken seriously in suicide prevention measures.

The present study demonstrated similar determinants of adolescent suicide attempts as the established risk factors from developed countries and individual LMICs, including poor socioeconomic status[12], bullying[45,46], mental problems[47,48], substance use[15,49], and weak family and social relationships[50]. Although considerable researches have highlighted the characteristics of adolescent suicide attempts, our understanding of many aspects of suicide attempts in adolescents remains fragmented. We should focus more on the functions of suicide attempts. For example, it is essential to make a distinction between factors associated with thoughts of suicide (e.g., stressful life events appraised as being humiliating or defeating) and those that increase the likelihood of translating such thoughts into actual suicidal behavior (e.g., low levels of social support) [51].

Based on the universal risk factors of adolescent suicide attempts, screening programs were implemented to identify adolescents at high risk of suicide attempts in school settings. Some evidence exists that school-based suicide prevention interventions can effectively reduce suicide attempts among adolescents in rich countries[52,53], however little is known about the effective interventions to reduce adolescent suicidal behaviors in low- and middle-income countries. More research regarding intervention strategies for suicide attempts should be conducted in LMICs.

The strength of our study is that we included a large sample size of GSHS data from 40 LMICs. Moreover, the GSHS relies on standardized sample selection procedures and standard measures of suicidal behaviors and risk factors, which make the results comparable between countries. However, our findings should be viewed with caution given the study limitations. First, the suicide attempts were self-reported, which might not reflect the true prevalence. Although studies have suggested the acceptable reliability and validity of self-reported suicidal behaviors among adolescents in rich countries[54,55], the measures do not appear to have been tested in LMICs. Second, data were collected over several years between 2009 and 2013, and the comparisons of prevalence between different countries should be made cautiously. Finally, the GSHS is limited to youth attending school, however according to the UNESCO Institute for Statistics, the rates of out-of school among LMICs are very high. Especially, among low-income countries, the lower secondary out-of-school is 38%, and the upper secondary out-of-school rate is 59%[56]. This imply that further study is needed among the out-of-school adolescents in LMICs.

In conclusion, we found that suicide attempts remain a major public health issue among young adolescents in LMICs. We also indicate that girls and older adolescents tend to make suicide attempts with a plan. Although our study has substantiated some universal risk factors of adolescent suicide attempts, further research is needed to understand the functions of suicide attempts as well as the intervention strategies to reduce the burden of suicidal behavior effectively.

## Supporting information

S1 Table Prevalence of suicide attempts in young adolescents by gender and country. (PDF)

S2 Table Prevalence of suicide attempts in young adolescents by age group. (PDF)
