# Supplementary material for: Prevalence, distribution, and associated factors of suicide attempts in young adolescents: School-based data from 40 low-income and middle-income countries
Source: PLoS One. 2018 Dec 19;13(12):e0207823. doi: 10.1371/journal.pone.0207823 (PMC6300318; doi:10.1371/journal.pone.0207823)
Supplement: S1 Table — (PDF) [file pone.0207823.s001.pdf]

S1 Table. Prevalence of suicide attempts in young adolescents by gender and country

Country	Total	Boys	Girls
**Africa**			
Benin	28.2(23.6-33.3)	28.2(23.3-33.8)	28.0(23.4-33.2)
Ghana	26.4(22.5-30.8)	25.5(21.5-30.0)	27.4(22.9-32.4)
Malawi	11.1(6.5-18.1)	11.4(6.4-19.4)	10.7(6.1-18.2)
Mauritania	16.9(11.7-23.8)	17.2(11.5-24.9)	16.6(11.7-22.9)
Namibia	25.6(21.8-29.8)	27.1(22.8-31.9)	24.2(20.5-28.4)
Swaziland	16.2(13.9-18.7)	16.3(13.5-19.5)	16.1(13.8-18.8)
**Americas**			
Antigua and Barbuda	12.3(10.3-14.7)	9.0(6.8-11.9)	15.8(12.1-20.2)*
Argentina	16.1(14.9-17.3)	13.8(12.1-15.7)	18.2(16.5-20.0)*
Bahamas	14.0(11.8-16.5)	13.2(9.9-17.2)	14.7(12.3-17.5)
Belize	13.3(11.3-15.5)	11.8(9.9-13.9)	14.7(11.9-18.0)
Bolivia	20.7(18.4-23.2)	16.5(13.7-19.7)	25.1(22.9-27.4)*
British Virgin Islands	12.4(10.9-14.1)	9.9(7.9-12.3)	14.8(12.5-17.3)*
Costa Rica	8.5(7.5-9.5)	6.5(5.5-7.6)	10.5(9.1-12.0)*
Dominica	15.0(12.9-17.4)	14.1(11.1-17.8)	15.9(13.3-18.9)
Guatemala	13.4(12.2-14.6)	10.4(8.7-12.3)	16.7(14.8-18.7)*
Honduras	17.2(15.1-19.7)	11.7(9.6-14.2)	22.2(18.8-26.0)*
Jamaica	24.1(18.7-30.5)	25.8(18.6-34.5)	22.3(17.3-28.2)
Peru	17.3(15.6-19.0)	12.5(10.7-14.4)	22.1(19.7-24.7)*
Saint Kitts and Nevis	13.5(13.5-13.5)	14.3(14.3-14.3)	12.6(12.6-12.6)
Salvador	13.1(11.2-15.2)	9.3(7.0-12.2)	17.1(14.4-20.1)*
Suriname	9.8(8.4-11.4)	6.3(4.7-8.3)	13.3(11.2-15.7)*
Trinidad and Tobago	13.5(11.9-15.4)	12.1(9.9-14.6)	14.9(12.3-18.0)
Uruguay	10.2(8.8-11.8)	8.1(6.6-10.0)	11.9(10.4-13.8)*
**Eastern Mediterranean**			
Iraq	16.1(13.8-18.6)	14.8(12.4-17.5)	17.8(14.0-22.4)
Kuwait	17.3(14.8-20.1)	16.4(12.7-20.9)	18.3(16.7-20.1)
Lebanon	13.6(12.1-15.3)	13.2(11.1-15.6)	14.0(11.2-17.5)
Morocco	13.9(11.8-16.3)	12.7(10.5-15.2)	15.4(12.8-18.4)*
Palestine	21.6(20.0-23.2)	22.5(20.4-24.7)	20.6(18.8-22.6)
United Arab Emirates	13.9(11.9-16.1)	14.0(12.1-16.1)	13.8(11.1-17.1)
**Asia**			
Cambodia	6.8(5.9-7.8)	5.7(4.4-7.4)	8.0(6.8-9.3)*
Malaysia	6.7(6.2-7.4)	6.3(5.6-7.2)	7.2(6.4-8.0)
Mongolia	9.8(8.8-11.0)	8.2(7.0-9.6)	11.3(9.9-12.9)*
Philippines	12.8(11.3-14.5)	11.1(9.1-13.5)	14.5(12.6-16.6)*
China[#]	5.9(5.4-6.4)	5.2(4.5-5.8)	6.7(5.9-7.4)*
**Western Pacific**			
Kiribati	31.5(28.4-34.7)	31.3(27.3-35.6)	31.6(28.2-35.2)
Niue	10.0(5.9-16.3)	10.5(5.3-19.9)	9.1(4.1-19.2)
Samoa	61.2(54.8-67.3)	67.8(61.1-73.9)▲	55.2(48.2-62.0)

Solomon Islands	33.6(26.2-42.0)	33.2(25.6-41.7)	34.2(25.8-43.7)
Tuvalu	8.2(6.6-10.3)	13.4(10.4-17.0)▲	3.5(2.1-5.6)
Vanuatu	24.4(19.7-29.9)	29.3(24.0-35.2)▲	19.3(13.9-26.3)

Data are from a large sample size study performed in 2010 in China

* *P*<0.05 for the difference between genders (girls > boys)

▲ *P*<0.05 for the difference between genders (boys>girls)

Data are expressed as prevalence rate (95%*CI*)
