# Supplementary material for: Prevalence, distribution, and associated factors of suicide attempts in young adolescents: School-based data from 40 low-income and middle-income countries
Source: PLoS One. 2018 Dec 19;13(12):e0207823. doi: 10.1371/journal.pone.0207823 (PMC6300318; doi:10.1371/journal.pone.0207823)
Supplement: S2 Table — (PDF) [file pone.0207823.s002.pdf]

S2 Table. Prevalence of suicide attempts in young adolescents by age group

Country	Total	12-13 years	14-15 years	16 years or older
**Africa**				
Benin	28.2(23.6-33.3)	29.0(19.6-40.7)	28.8(23.4-34.8)	27.5(23.1-32.5)
Ghana	26.4(22.5-30.8)	24.0(19.1-29.7)	27.0(20.9-34.0)	26.9(23.0-31.2)
Malawi	11.1(6.5-18.1)	10.0(4.9-19.2)	11.3(6.5-19.0)	26.6(6.6-64.8)
Mauritania	16.9(11.7-23.8)	15.8(9.3-25.6)	17.9(12.4-25.0)	16.0(10.2-24.3)
Namibia	25.6(21.8-29.8)	24.5(18.4-31.7)	26.7(22.0-32.2)	25.2(21.4-29.4)
Swaziland	16.2(13.9-18.7)	11.5(8.3-15.8)	16.3(13.2-19.9)*	16.9(14.6-19.4) *
**Americas**				
Antigua and Barbuda	12.3(10.3-14.7)	11.7(8.2-16.4)	12.5(9.9-15.7)	15.8(3.6-48.7)
Argentina	16.1(14.9-17.3)	14.6(12.2-17.2)	16.4(14.8-18.1)	17.2(15.3-19.2)
Bahamas	14.0(11.8-16.5)	12.5(10.0-15.5)	14.9(11.9-18.6)	22.0(14.3-32.1) *
Belize	13.3(11.3-15.5)	9.8(7.6-12.5)	14.0(11.6-16.8) *	19.0(13.9-25.5) *
Bolivia	20.7(18.4-23.2)	18.1(14.5-22.5)	20.8(18.7-23.1)	23.3(18.9-28.3) *
British Virgin Islands	12.4(10.9-14.1)	10.4(8.1-13.3)	13.5(11.1-16.4)	13.4(10.4-17.2)
Costa Rica	8.5(7.5-9.5)	8.8(7.1-10.9)	7.6(6.2-9.2)	10.7(8.6-13.3)
Dominica	15.0(12.9-17.4)	11.7(9.1-14.9)	15.7(13.1-18.7) *	19.4(14.6-25.3) *
Guatemala	13.4(12.2-14.6)	12.9(10.1-16.5)	13.6(11.6-15.9)	13.2(10.7-16.2)
Honduras	17.2(15.1-19.7)	18.8(16.2-21.7)	15.7(12.6-19.2)	17.8(13.8-22.7)
Jamaica	24.1(18.7-30.5)	26.5(17.6-37.8)	21.7(16.1-28.5)	27.7(18.8-38.8)
Peru	17.3(15.6-19.0)	16.2(13.3-19.6)	17.4(15.2-19.9)	18.0(14.2-22.5)
Saint Kitts and Nevis	13.5(13.5-13.5)	13.8(13.8-13.8)	13.9(13.9-13.9) *	11.7(11.7-11.7)
Salvador	13.1(11.2-15.2)	10.4(7.6-14.1)	13.0(10.8-15.5)	18.7(14.4-23.9) *
Suriname	9.8(8.4-11.4)	5.9(4.1-8.5)	8.4(6.7-10.5) *	13.3(10.8-16.4) *
Trinidad and Tobago	13.5(11.9-15.4)	9.5(8.3-10.8)	16.0(13.3-19.2) *	17.7(13.5-22.8) *
Uruguay	10.2(8.8-11.8)	6.0(4.4-8.0)	10.4(8.7-12.5) *	15.0(12.4-18.1) *
**Eastern Mediterranean**				
Iraq	16.1(13.8-18.6)	12.3(9.8-15.5)	15.3(12.8-18.3)	21.7(16.7-27.7) *
Kuwait	17.3(14.8-20.1)	16.1(13.1-19.6)	16.4(13.4-19.9)	24.0(17.8-31.4) *
Lebanon	13.6(12.1-15.3)	10.8(9.3-12.5)	14.6(12.7-16.8) *	19.1(14.9-24.1) *
Morocco	13.9(11.8-16.3)	11.1(9.4-13.1)	14.3(11.7-17.2) *	19.0(15.5-23.1) *
Palestine	21.6(20.0-23.2)	21.5(18.9-24.3)	21.2(18.7-23.9)	29.3(23.8-35.4) *
United Arab Emirates	13.9(11.9-16.1)	12.1(9.4-15.4)	12.5(10.1-15.4)	25.9(20.7-31.9) *
**Asia**				
Cambodia	6.8(5.9-7.8)	4.9(2.7-8.8)	8.0(6.5-9.9)	6.4(5.4-7.5)
Malaysia	6.7(6.2-7.4)	6.6(5.8-7.5)	7.2(6.3-8.2)	6.3(5.7-7.0)
Mongolia	9.8(8.8-11.0)	9.4(8.1-10.9)	9.5(8.1-11.1)	10.6(8.3-13.3)
Philippines	12.8(11.3-14.5)	11.1(7.9-15.4)	13.3(11.8-14.9)	13.7(11.7-15.9)
**Western Pacific**				
Kiribati	31.5(28.4-34.7)	27.5(22.3-33.3)	32.2(28.6-35.9)	35.6(28.4-43.5)
Niue	10.0(5.9-16.3)	10.3(3.9-24.9)	12.0(5.0-26.2)	8.0(3.3-18.0)
Samoa	61.2(54.8-67.3)	62.4(54.3-69.9)	59.9(52.8-66.5)	70.9(58.1-81.0)

Solomon Islands	33.6(26.2-42.0)	24.8(16.5-35.6)	34.7(26.2-44.3) *	37.6(28.9-47.2) *
Tuvalu	8.2(6.6-10.3)	10.1(7.4-13.8)	8.7(5.9-12.6)	4.8(2.7-8.6)*
Vanuatu	24.4(19.7-29.9)	24.8(16.8-34.9)	22.6(18.2-27.8)	28.5(20.5-38.2)

* $P<0.05$ for the difference between adolescents aged 12-13 years and the other age group.

Data are expressed as prevalence (95%*CI*)
